# On Cod-Liver Oil in Phthisis

**Published:** 1851-01

**Authors:** 


*On Cod-Liver Oil in Phthisis.* M. Duclos thus sums up the results of his experience with this substance:

1. The presence of fever is what we must chiefly attend to, relying more on this remedy when it is absent, and less when it is present.
2. The remedy frequently arrests the progress of the disease when only in the first stage.
3. It rarely arrests it when in the second stage, although it may retard it.
4. The third stage is not favorably influenced by the oil.
5. The oil should be administered for a considerable time; and, if a good effect results, it should be suspended awhile, to be again resumed. Thus, it may be given for two months, and then suspended for a fortnight, resumed for a month, and re-suspended for a fortnight again, so as gradually to reduce the length of the intervals during which it is given.
6. The clear, slightly smelling, nearly tasteless oil, is less efficacious than the brown, thick, strong oil.—*Brit. and For. Med.-Chir. Rev.*, October, 1850, from *Bull. de Thér.*—*Med. Jour. Phar.*

